# Supplementary material for: Distinct Gut Microbiota Signatures Are Associated with Severity of Metabolic Dysfunction-Associated Steatotic Liver Disease in People with HIV
Source: Int J Mol Sci. 2025 Aug 22;26(17):8165. doi: 10.3390/ijms26178165 (PMC12428781; doi:10.3390/ijms26178165)
Supplement: Supplementary file 1 [file ijms-26-08165-s001.zip › ijms-3810167-supplementary.pdf]

Supplementary Figure S1. Correlation analysis showing the association between expression of dihydroneopterin triphosphate diphosphatase (*K08310)* and liver stiffness between PWH with and without severe MASLD.

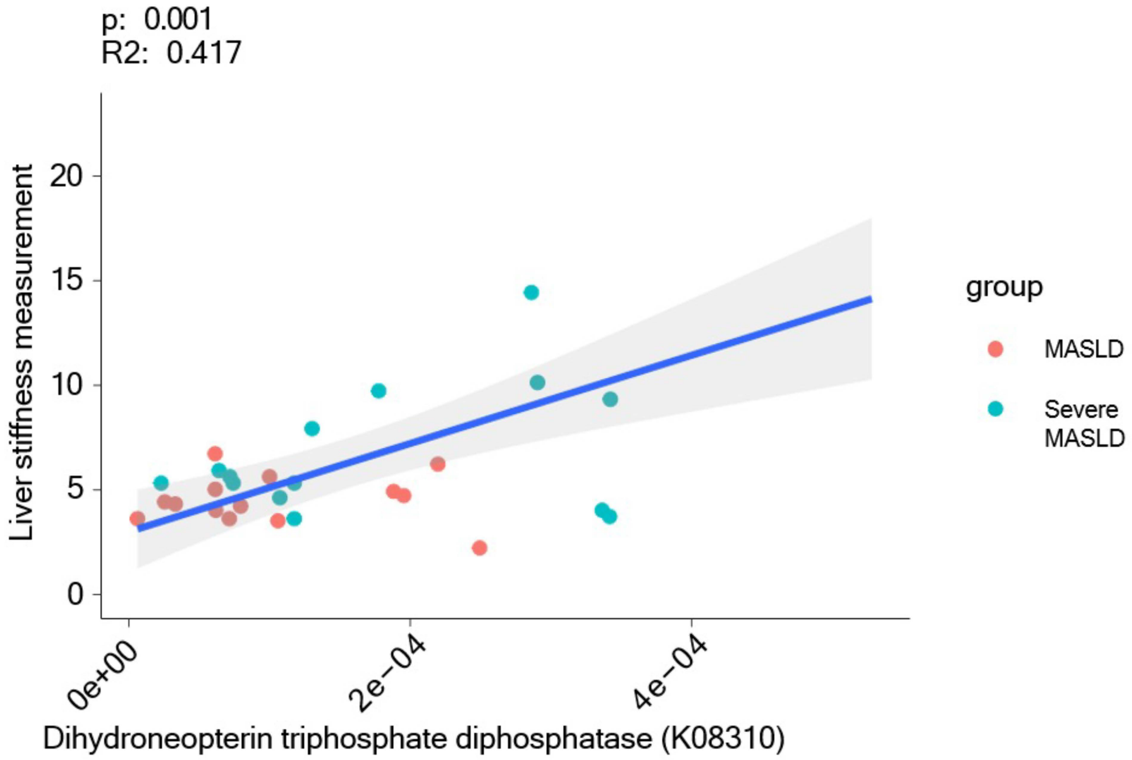

Supplementary Figure S2. Correlation analysis showing the association between expression of 7-cyano-7-deazaguanine reductase (K06879) (Fig. S2a), succinate semialdehyde dehydrogenase (K08324) (Fig. S2b), 3-hydroxyacyl-CoA dehydrogenase/enoyl-CoA hydratase/3-hydroxybutyryl-CoA epimerase/enoyl-CoA isomerase (K01825) (Fig. S2c), GTP-cyclohydrolase I (K01497) (Fig. S2d), erythron-7,8-dihydroneopterin-triphosphate epimerase (K07589) (Fig. S2e), and dihydromonapterin reductase/dihydrofolate reductase (Fig. S2f) and serum triglycerides between PWH with and without severe MASLD.

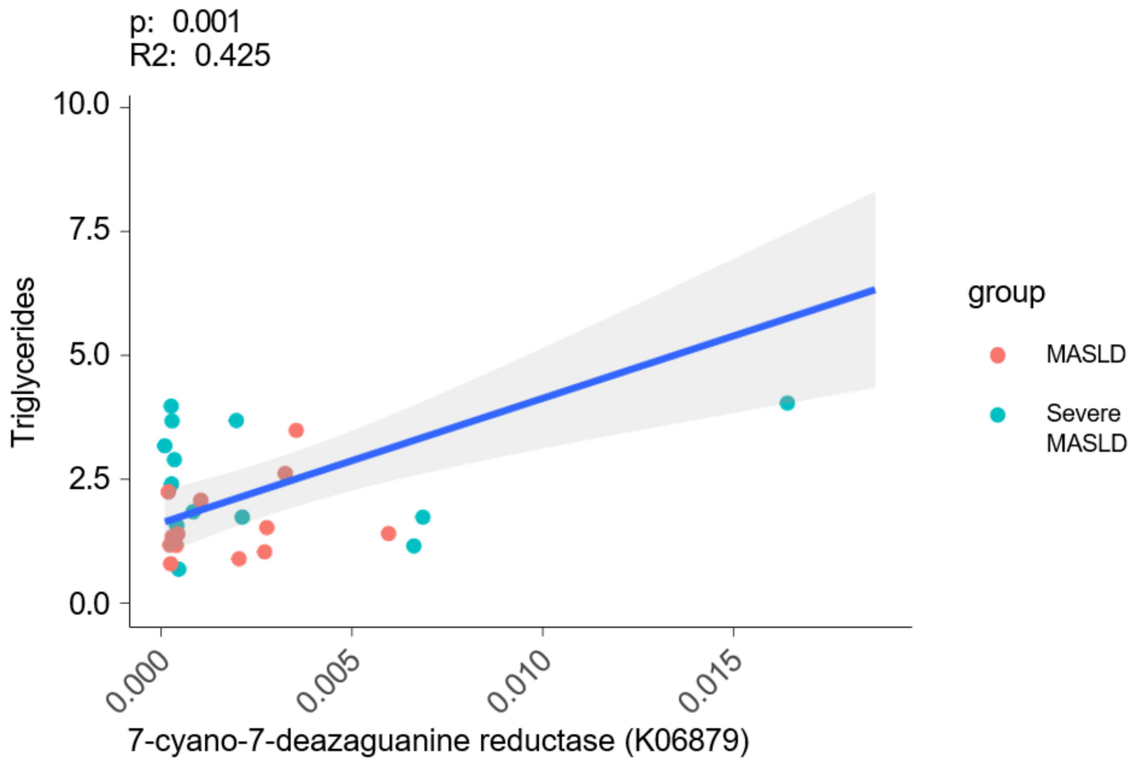

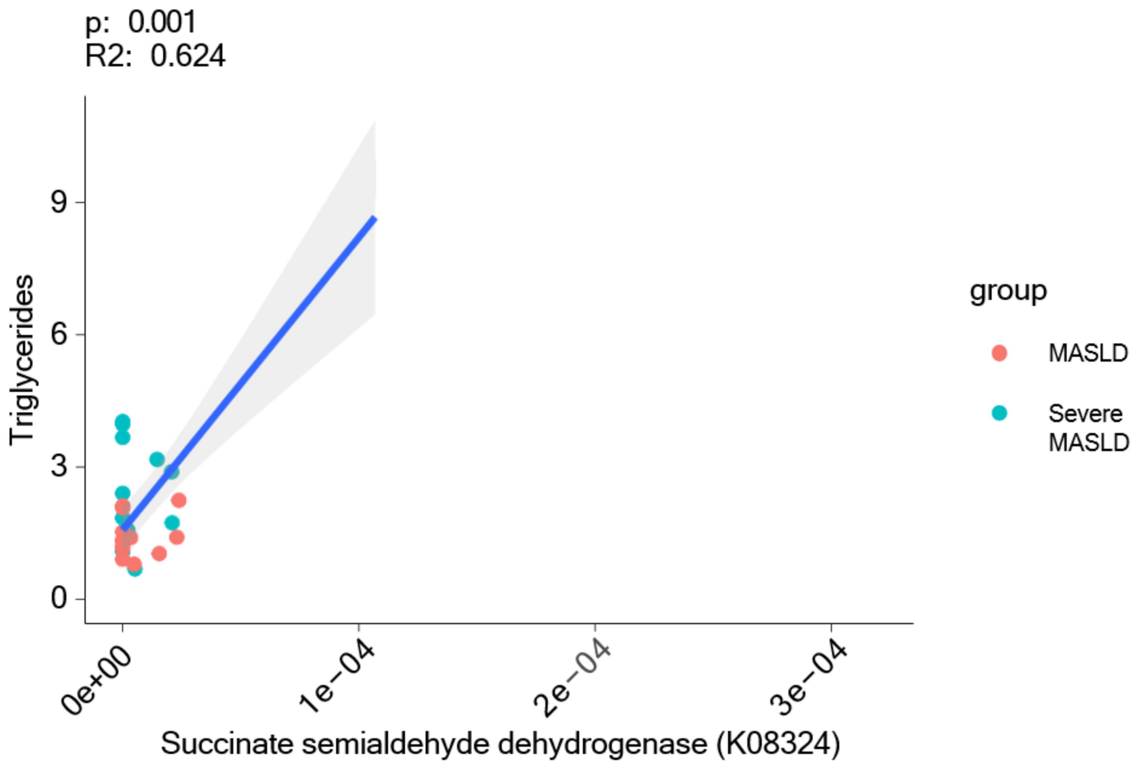
p: 0.001
R2: 0.624
Triglycerides
0
3
6
9
0e+00
1e−04
2e−04
3e−04
Succinate semialdehyde dehydrogenase (K08324)
group
MASLD
Severe MASLD

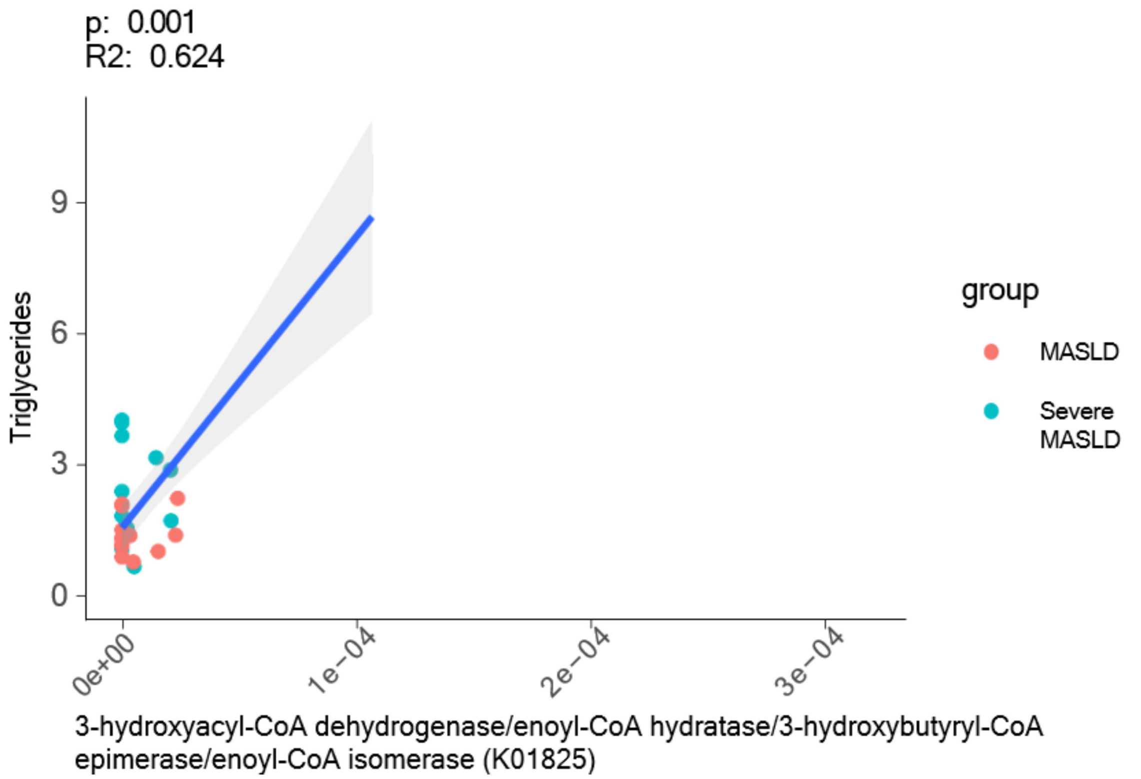
p: 0.001
R2: 0.624
Triglycerides
0
3
6
9
0e+00
1e−04
2e−04
3e−04
3-hydroxyacyl-CoA dehydrogenase/enoyl-CoA hydratase/3-hydroxybutyryl-CoA epimerase/enoyl-CoA isomerase (K01825)
group
MASLD
Severe MASLD

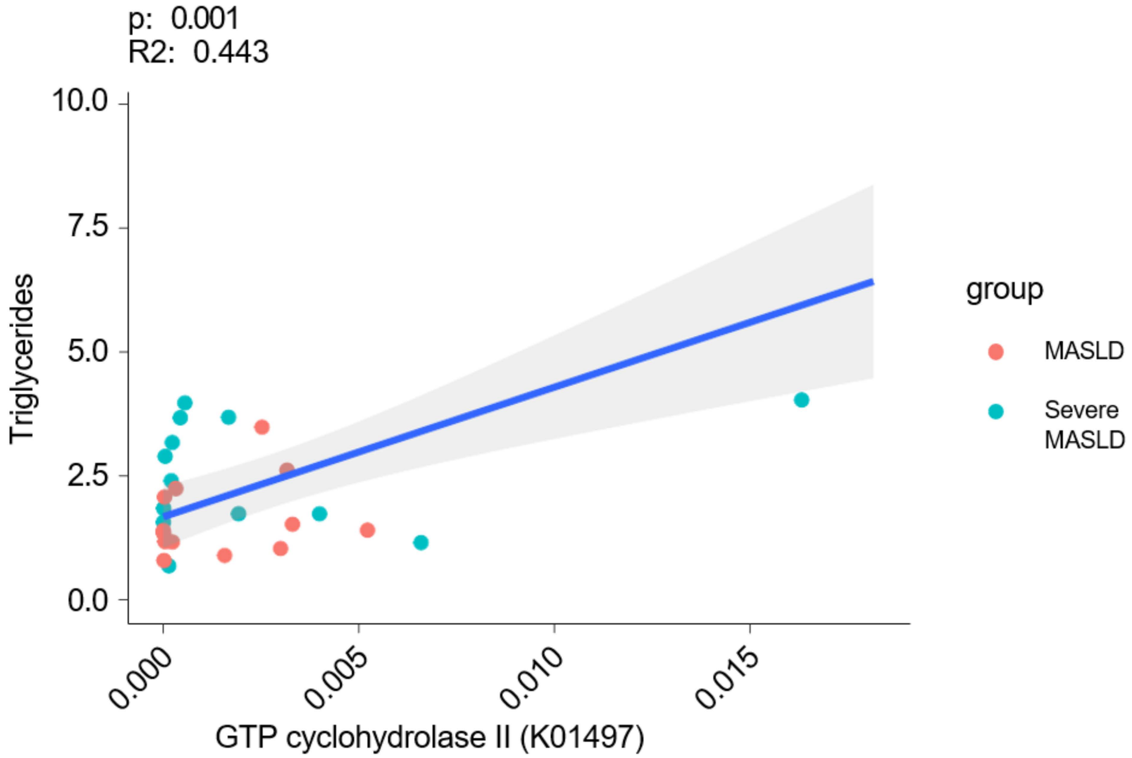
p: 0.001
R2: 0.443
Triglycerides
10.0
7.5
5.0
2.5
0.0
0.000
0.005
0.010
0.015
GTP cyclohydrolase II (K01497)
group
MASLD
Severe MASLD

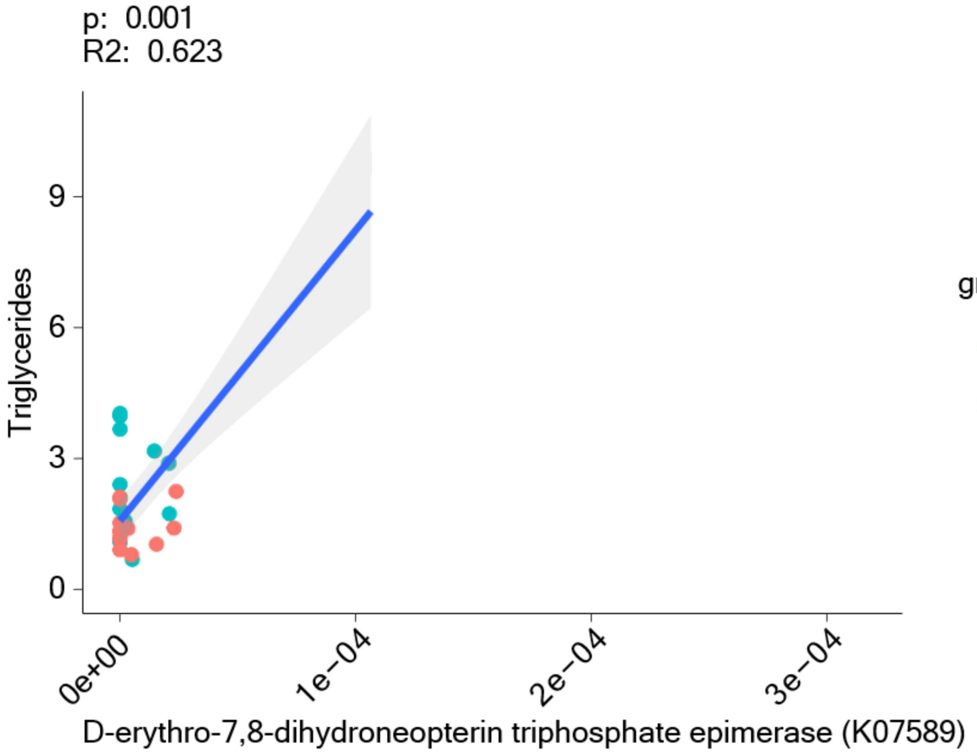
p: 0.001
R2: 0.623
Triglycerides
9
6
3
0
0e+00
1e−04
2e−04
3e−04
D-erythro-7,8-dihydroneopterin triphosphate epimerase (K07589)
group
MASLD
Severe MASLD

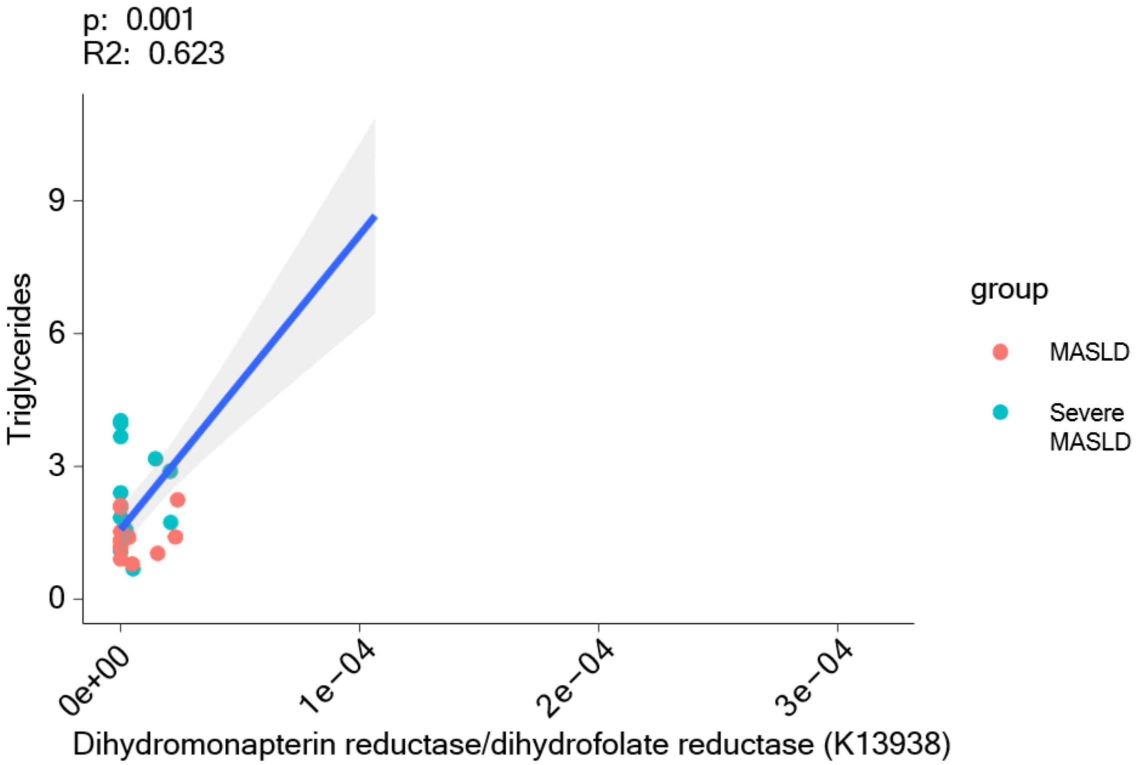
p: 0.001
R2: 0.623
Triglycerides
9
6
3
0
0e+00
1e−04
2e−04
3e−04
Dihydromonapterin reductase/dihydrofolate reductase (K13938)
group
MASLD
Severe MASLD

Supplementary Figure S3. Correlation analysis showing the association between abundance of the bacterial genera *Holdemanella* (Fig. S3a) and *Escherichia-Shigella* (Fig. S3b) and serum triglycerides between PWH with and without severe MASLD.

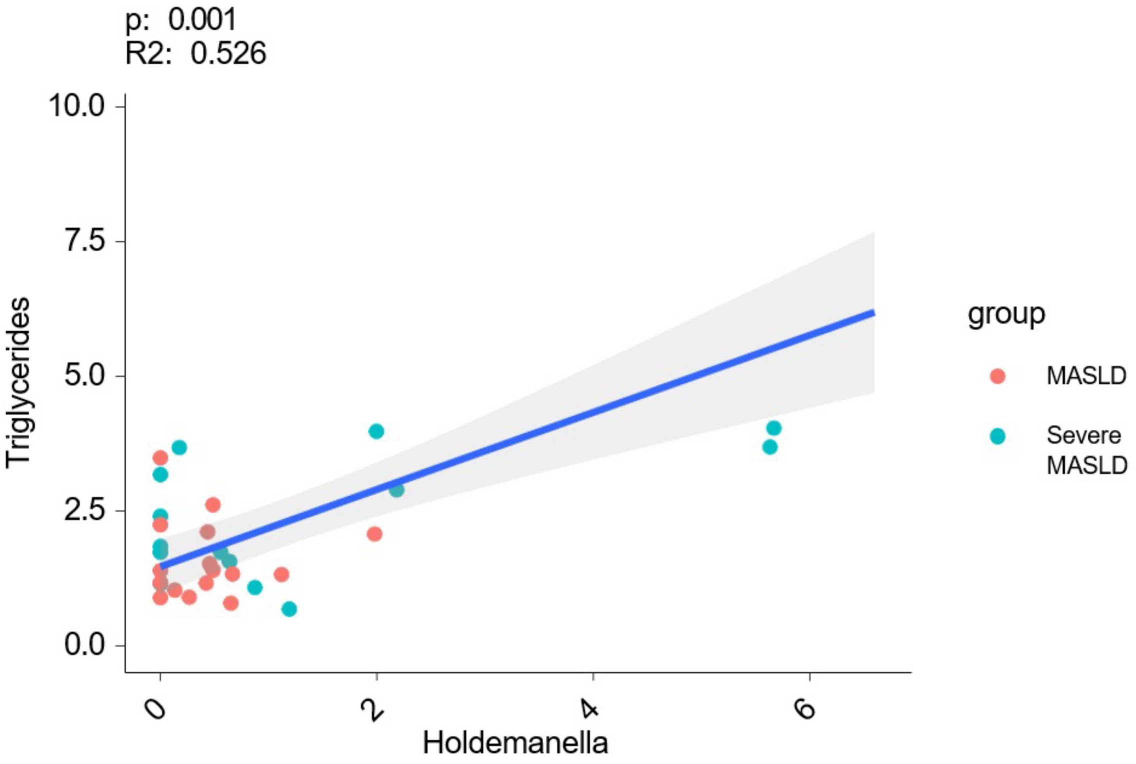

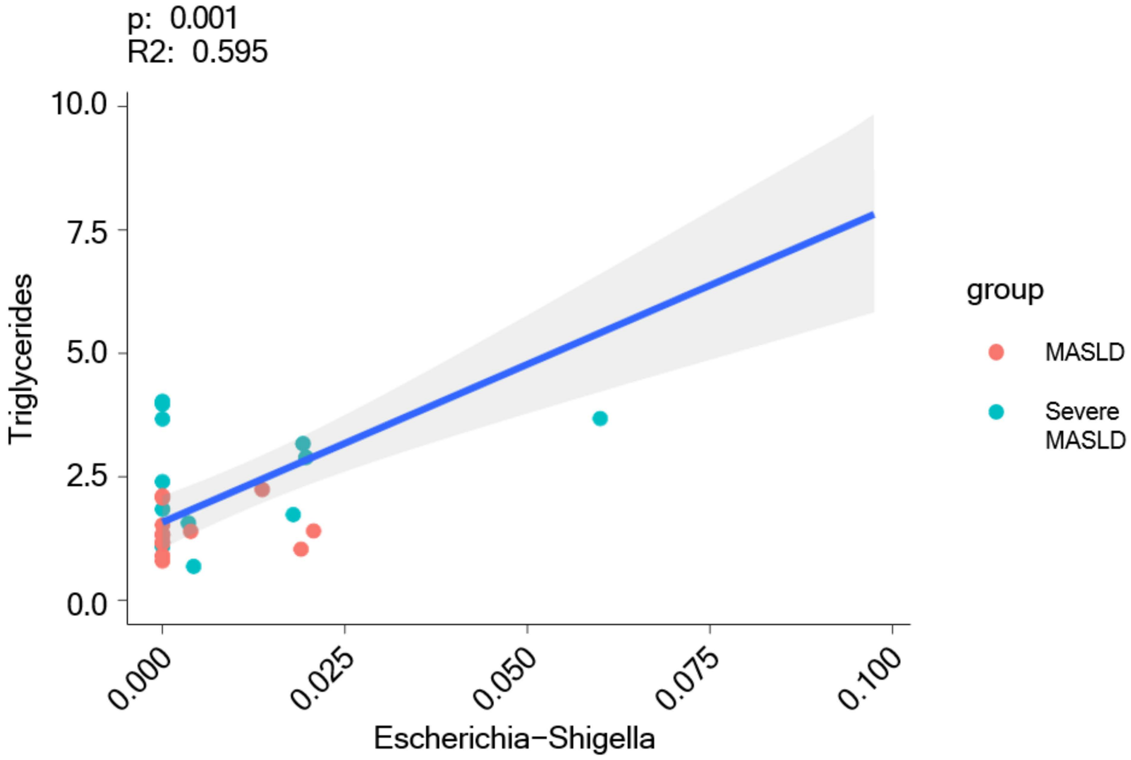
p: 0.001
R2: 0.595
10.0
7.5
5.0
2.5
0.0
Triglycerides
0.000
0.025
0.050
0.075
0.100
Escherichia−Shigella
group
MASLD
Severe
MASLD
